# Estimating the causal effects of income on health: how researchers’ definitions of “income” matter

**DOI:** 10.1186/s12889-024-19049-w

**Published:** 2024-06-11

**Authors:** Erik Igelström, Daniel Kopasker, Peter Craig, Jim Lewsey, Srinivasa Vittal Katikireddi

**Affiliations:** 1 MRC/CSO Social and Public Health Sciences Unit, School of Health and Wellbeing, University of Glasgow, Glasgow, UK; 2 Health Economics and Health Technology Assessment, School of Health and Wellbeing, University of Glasgow, Glasgow, UK

**Keywords:** Income, Health, Causal inference, Income change; Social epidemiology

## Abstract

**Background:**

There is a well-established cross-sectional association between income and health, but estimates of the causal effects of income vary substantially. Different definitions of income may lead to substantially different empirical results, yet research is often framed as investigating “the effect of income” as if it were a single, easily definable construct.

**Methods/Results:**

The aim of this paper is to introduce a taxonomy for definitional and conceptual issues in studying individual- or household-level income for health research. We focus on (1) the definition of the income measure (earned and unearned; net, gross, and disposable; real and nominal; individual and household; relative and absolute income) and (2) the definition of the causal contrast (amount, functional form assumptions/transformations, direction, duration of change, and timing of exposure and follow-up). We illustrate the application of the taxonomy to four examples from the published literature.

**Conclusions:**

Quantified estimates of causal effects of income on health and wellbeing have crucial relevance for policymakers to anticipate the consequences of policies targeting the social determinants of health. However, much prior evidence has been limited by lack of clarity in distinguishing between different causal questions. The present framework can help researchers explicitly and precisely articulate income-related exposures and causal questions.

## Introduction

Socioeconomic conditions have a large influence on health and wellbeing, and the social determinants of health are a major focus for both public health research and policymaking [1, 2]. In particular, there is a well-established association between income and many health outcomes, and evidence that changes in income can change health [3–6]. However, reported estimates of the causal effects of income on health vary substantially.

Different definitions of income (for example, individual versus household income) may lead to substantially different empirical results [7]. Despite this, many studies have been framed as investigating “the effect of income” as if it were a single, easily definable construct, without recognising these nuances and their implications for the generalisability and transferability of results. This makes it difficult to understand whether heterogeneity between studies reflects genuine differences between populations or contexts, or merely different methodological and definitional choices.

**Fig. 1:**
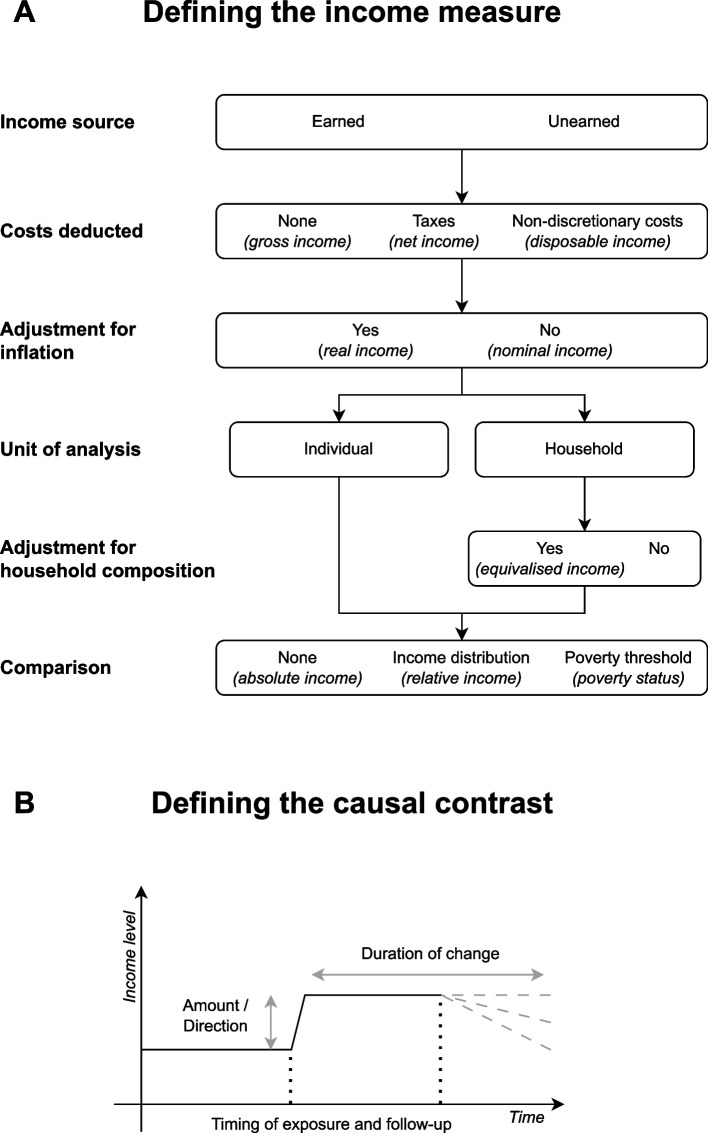
Visual overview of key definitional and conceptual issues in studying individual- or household-level income as an epidemiological exposure

To address this difficulty, we present a taxonomy for definitional and conceptual issues to consider when studying income as an epidemiological exposure, and discuss their implications in terms of psychosocial and material pathways from income to health [8]. Our discussion of these issues is structured around (1) how income is measured, and (2) how the causal contrast is defined (Fig. 1). We illustrate the use of this taxonomy by applying it to four published studies. We limit the scope to individual- or household-level income; hence, we are not considering the effects of area-level income characteristics on both individual and area-level health.

### Defining the income measure

The first issue to consider is how income is being defined. Differently defined measures of income are interrelated but not interchangeable, and different types of income are likely to affect health in different ways. It is in this respect similar to many other epidemiological variables, where related variables may be used for similar purposes, but cannot be treated interchangeably. In practice, choices about what measure to use are often dictated by the nature and limitations of the data used, rather than deliberately made by the researcher. This may limit the kinds of questions that can be asked, and it is essential that researchers adopt an appropriate interpretation of results given the available income measures.

### Income source

Income data are often disaggregated by income source. It is common to distinguish between *earned* and *unearned income*. Earned income encompasses income obtained through the supply of labour, e.g., salaries or wages from employment, or income from business activities or self-employment. Unearned income in principle encompasses all other sources, including government benefits, income from investments or property (such as interest, dividends, rent, and capital gains), retirement income, inheritances, lottery winnings, and gifts. The nature of the data source will also affect what income sources are captured: for example, tax register data may only encompass reported taxable income, and thus omit informal, illegal, non-monetary or otherwise unrecorded receipts. When income is self-reported, the context and nature of the question asked will also affect the income sources a respondent considers and the accuracy of their response.

Neoclassical economic theory generally assumed that money is a fungible resource, and hence that a rational person would make the same decisions in response to a given amount of income regardless of the source. However, this is often not the case in reality: the “mental accounting” processes that underlie economic decisions are now appreciated to be more complex [9]. For example, if a cash transfer is labelled as being for a specific purpose, it may be more likely to be used for that purpose [10], and windfall income may be spent differently from regular or expected income [9]. The practical upshot of this is that the causal effects of two different interventions on income might differ depending on the type of income targeted, even if the amounts are identical.

### Costs deducted: gross, net, and disposable income

A person’s *gross income* is their total income from all sources, prior to taxes being deducted. For many material pathways to health, such as a person’s ability to buy nutritious food or engage in leisure activities, what matters is not necessarily gross income, but how much of it is available to spend.

**Fig. 2:**
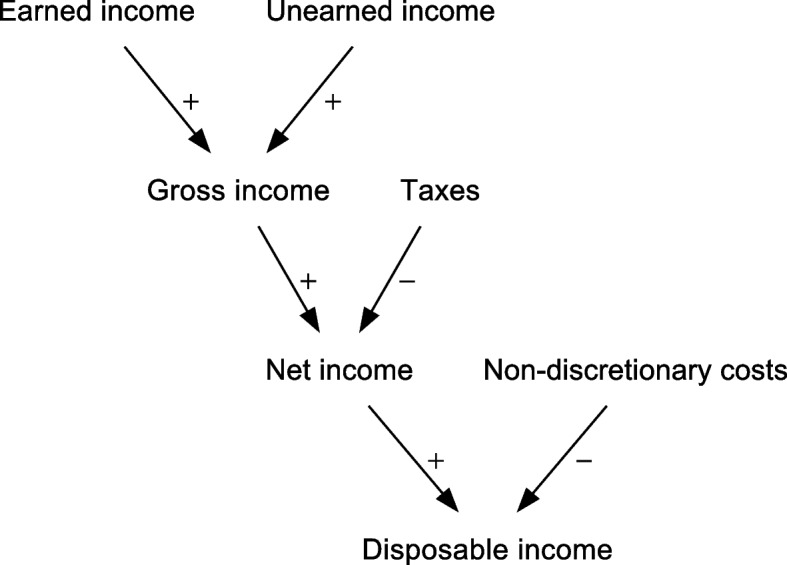
Illustration of how income measures can be constructed by combining or subtracting other measures

*Net income* refers to income after direct taxes have been subtracted (Fig. 2). *Disposable income* is what remains of the disposable income after subtracting *non-discretionary costs*, which may be defined differently in different contexts and data sources. Non-discretionary costs generally include housing costs (rent or mortgage repayments), and may also include other costs that can be considered necessary or unavoidable: for example, repayments of non-housing debt (including student debt), utilities, food, transport, healthcare, and clothing (sometimes including for dependents). All other costs are considered discretionary. Although these terms seem to imply a normative judgement, the distinction is typically drawn in a coarse and arbitrary way, and does not necessarily reflect what individuals in a given context genuinely consider unavoidable or dispensable [11]. They are thus often best viewed as purely technical terms, whose precise meaning needs to be specified.

### Adjustment for inflation: real and nominal income

When comparing amounts of money over time, it is generally necessary to consider *inflation* – that is, the rate at which the prices of goods and services change over time. Measures of *real income* have been adjusted for inflation, such that a change reflects a genuine change in “purchasing power” (i.e., the amount of goods and services that can be obtained with a given amount). Real amounts are generally expressed in terms of the equivalent monetary amount in some given baseline year (e.g. “2010 US dollars”). The unadjusted amount is called *nominal income*. When comparing incomes across multiple time points, it is typically more appropriate to use a measure of real rather than nominal income.

Real or inflation-adjusted amounts are calculated with reference to a price index, which may be based on macroeconomic measures such as gross domestic product or the price of a fixed set of goods and services [12]. While these measures describe general trends in price changes, the extent of inflation is often different across different goods and services, and the impact can differ across population groups: for example, increases in food and energy prices may affect low-income households more than high-income households [13].

### Unit of analysis: individual or household

An individual’s own personal income is not always the best way to capture the financial resources they have access to: for example, people may rely on the income of their spouses, parents, children, or others in addition to their own. For this reason, it is often useful to aggregate income by household or some other group. A household is typically defined as a group of people who live in the same dwelling, but more complex definitions may be appropriate depending on the context [14].

The concept of a household is necessarily simplistic, and does not account for more complex family relationships, such as shared care for children between multiple households. Because of the gendered distribution of both labour market participation and wages, the discrepancy between individual and household income measures is often greater for women [7]. It is important to consider how the choice of income measure in a study may affect different populations, particularly when stratifying by gender.

### Adjustment for household composition

When comparing incomes across households, household size and composition need to be taken into account, since higher income in a larger household may be offset by higher costs. Since these costs do not increase uniformly for each additional person, simply calculating the per capita income is likely to be misleading; instead, equivalisation scales are often used to calculate an *equivalised household income* based on the number and ages of household members. Multiple such scales are in use [15]; Table 1 illustrates how equivalised household income is calculated using the modified OECD scale [16]. Although such standard equivalence scales are widely used, their validity and accuracy in a specific time and place are rarely tested or justified; they may not necessarily reflect true cost differences experienced by households, which are of course highly context-dependent.

**Table 1:** Illustration of how equivalised household income is calculated using the modified OECD scale

	Equivalisation factor^a^	Total household income	Equivalised household income^b^
First adult in the household	1.0		
Each additional household member aged 14 + years	0.5		
Each additional household member aged 0–13 years	0.3		
**Example calculations**			
One adult	1.0	20,000.00	20,000.00
Two adults	1.5	20,000.00	13,333.33
One adult, two children aged 0–13 years	1.6	20,000.00	12,500.00
Two adults, two children aged 0–13 years	2.1	20,000.00	9,523.81

^a^Based on the "modified OECD scale"; Hagenaars A, de Vos K, Zaidi MA. Poverty statistics in the late 1980s: research based on micro-data. Luxembourg: Office for Official Publications of the European Communities; 1994

^b^Equivalised household income is calculated by dividing the total household income by the equivalisation factor

### Comparison: absolute and relative income

Income can either be measured in absolute terms (i.e., in units of currency) or in relative terms compared to the income distribution in some reference group (for example, income rank or percentile). It has been suggested that relative income, i.e., one’s actual or perceived position in the income distribution, may have an effect on some outcomes (particularly wellbeing) independently of absolute amount [17, 18]. Relative income can be expressed in terms of income quantiles (e.g., quintiles, deciles, or percentiles), or by employing a threshold defined in terms of the income distribution: for example, poverty is often defined as an income below some proportion of the median income. The categories defined by quantiles or poverty thresholds are necessarily somewhat arbitrary, and individuals just above or just below a threshold are likely to share a lot of characteristics. Hence it is worth noting that a change in poverty status or income quintile may sometimes represent only a small change in actual circumstances.

Relative and absolute measures of income capture substantially different things, and the difference is particularly relevant when the income distribution itself changes over time. For example, even a large increase in a household’s absolute income would not change that household’s position in the relative income distribution if household income increased by a similar percentage across the population. Whether this property is desirable or not depends entirely on the research question at hand.

### Defining the causal contrast

Following dominant practice in epidemiology and quantitative social science, we will assume that a causal effect has to be defined in terms of a *causal contrast* – intuitively, we must be able to answer the question “the effect of what, compared to what?” [19]. For a single intervention at a single point in time, the causal contrast is typically between the two potential outcomes where an individual received the intervention and where they did not. Since income varies continuously over a person’s life, the possibilities for defining different causal contrasts are much wider. For time-varying exposures, causal contrasts are typically conceived as comparing two different exposure regimens; i.e., well-defined sequences of exposures [20]. As with the choice of income measure, we will see that the choice of causal contrast can fundamentally affect which causal mechanisms are involved. In other words, the different causal contrasts implied by different study designs are not merely different strategies for estimating the same “true” causal effect of income, but instead often estimate substantially different effects.

For simplicity, we will mostly describe causal contrasts in this section in terms of *income changes*; i.e., increases or decreases that last for a certain length of time. Some prior literature has drawn a distinction between income change and income level, where differences in level represent persistent and often structural inequalities that may affect health in a distinct way [21]. By using this terminology, we do not mean to suggest that persistent differences in income level are unimportant or should not be regarded as causes. Rather, we propose that for the purpose of defining causal effects, thinking in terms of change promotes clarity. Comparing two individuals whose income level has differed throughout their lives is conceptually different from comparing two individuals whose income levels only recently diverged. Defining causal contrasts in terms of change emphasises the importance of specifying when the counterfactual scenarios diverge. We suggest that the distinction between “level” and “change” is not rigid, and is primarily a question of timescales: while the term “change” implies a short-term exposure and “level” a longer-term difference, both can be understood as referring to different exposure regimens.

### Amount, functional form, and transformations

The first feature of an income change that needs to be described is its size. In some settings, such as trials and policy evaluations, the intervention may be an income change of a specific amount. In others, individuals may be exposed to differently sized income changes, and we may want to infer a single effect estimate. We may also need to generalise from the observed changes what effect a differently sized change would have. In all these cases, we need to make assumptions about the functional form of the relationship.

The simplest functional form is a linear relationship, where a £1 change in income would always have the same average effect on health, and, say, a £10 change an effect 10 times as great. This would imply, for example, that a £500 increase in monthly income from £500 to £1,000 would have the same effect on health as an increase from £2,000 to £2,500, and that a £1,000 increase in either situation would have twice that effect. It is clear from both cross-sectional and longitudinal evidence that such a linear relationship is unlikely: additional income appears to make a greater difference to health at the lower end of the income scale [22]. Hence, it is usually necessary to apply some transformation to an income variable before using it as a predictor in a statistical model.

Perhaps the most commonly used transformation is the logarithm. A change in the logarithm of income (“log income”) represents a percentage change rather than a unit change: for example, an increase of 0.693 in log income corresponds to a doubling in income, regardless of whether this means a change from £100 to £200 or from £5,000 to £10,000. A 0.01 change in log income corresponds to approximately a 1% change in income, 0.02 to approximately 2%, and so forth; however, this rule of thumb becomes increasingly inaccurate at higher percentages.

The interpretations above are applicable when income is first log-transformed, and then the change in log income is calculated. Occasionally, changes in income are calculated first and then log-transformed. Importantly, this “log of change” is mathematically very different from the “change in log”, and cannot be interpreted as straightforwardly.

A well-known limitation of the log transformation is that it cannot be applied to zero or negative values. The inverse hyperbolic sine transformation (arsinh) is sometimes used as an alternative that does not have this limitation. Except for values very close to zero, a change in arsinh-transformed income is nearly identical to the equivalent change in log income, and can be interpreted in the same way.

In practice, visualising the relationship between the transformed or untransformed income variable and the outcome variable (for example, using a binned scatter plot) can be a useful way to assess whether a given transformation is reasonable. If a log or arsinh transformation is insufficient, more complex approaches can be used, including splines, fractional polynomials, or interactions with position in the income distribution. These may allow for more fine-grained or assumption-free analyses, but may also make the interpretation of numerical results more difficult. Reporting predicted probabilities or marginal effects may be more practically useful than regression coefficients.

Regardless of how the functional form of the income–health relationship is represented, we must be careful about generalising beyond the specific, observed circumstances of a study. For example, if a study sample only contained examples of income changes of 1–5%, the study is likely to be informative only about changes of a similar scale, unless we are prepared to make strong assumptions about the functional form beyond the observed values. Although we could, mathematically, report the results in terms of “the effect of a 10% change” (or even greater), we would not necessarily be justified in interpreting them as such.

### Direction of change

It may be important to distinguish between income gains and losses. Many analytical approaches rely on the assumption that the positive effect of an increase would be the same size as the negative effect of an equivalent decrease. However, this is unlikely to be true. The asymmetry of gains and losses is a key feature of prospect theory, which focuses on the behavioural responses to anticipated changes [23]. There is also evidence that income losses have a greater negative impact on health and wellbeing outcomes than the positive impact of income gains [6, 24, 25].

### Duration of change

It is also important to consider how long-lasting an income change is. A cash transfer scheme, for example, may have a limited duration (e.g., a single one-off payment, a 12-month period, etc.), or may last indefinitely. Clearly, this distinction becomes increasingly important when the outcome is measured some time after the onset of the exposure, since a longer-lasting payment would add up to a greater total amount. However, the anticipated duration may also be relevant for short-term outcomes. First, an income change that is expected to be temporary may be less beneficial for some mental health outcomes than one that is expected to be permanent. Second, expectations about future income can play a role in decision-making, potentially affecting health-related as well as economic behaviour. Models for explaining such decision-making include the literature on “time discounting” [26], which focuses on how future expectations affect trade-offs between immediate and delayed gains, and the “permanent income hypothesis”, which holds that consumption behaviour is primarily influenced by one’s expected long-term income rather than actual income in the short term [27].

Variation and insecurity of income over time may in itself be an important determinant of health [28]. Various measures of economic insecurity and precarity have been proposed, ranging from subjective measures (such as perceived job security or perceived ability to raise emergency funds when needed) to objective (such as recent experience of a substantial income drop) [29]. The concept of economic insecurity is inherently related not just to income, but also to other economic variables such as wealth and debt.

### Timing of exposure and follow-up

A related but distinct consideration is at what time the outcome is measured relative to the exposure. The true effect of an income change on an outcome measured after ten years may be different from the effect on the same outcome after one year. This may be because the relevant causal mechanisms take time to act, resulting in a delay, or it may be that effects are cumulative, and grow in size after prolonged exposure. For example, cancer mortality rates may take years or decades to respond to a change in income, if the main mechanisms involve changes in other environmental or behavioural exposures, which in turn affect incidence, and only subsequently mortality. On the other hand, mortality due to suicide has been observed to respond rapidly to changes in economic circumstances [30]. Even outcomes that respond quickly may also be partially mediated by slower mechanisms, in which case longer follow-up times would still be needed to capture the total long-term effects.

The timing of the income change itself during the life course is also important. Changes in household income impact child health outcomes differently and through different mechanisms than adult health [5]. Most of the available evidence on these impacts relates to health outcomes measured in children or adolescents, for obvious practical reasons: investigating the effect of income in childhood on health in adulthood requires a very long follow-up time. This may often not be practically feasible, and crucially increases the difficulty of drawing causal conclusions from non-experimental study designs.

## Further considerations

### Wealth and debt

Separately from income, a person’s financial resources can be measured in terms of wealth. Whereas income denotes the flow of resources received during some time period, wealth denotes the stock of resources owned at a point in time. Wealth can consist of monetary savings, investments such as stocks or bonds, or non-monetary assets such as land and property. Wealth can itself generate income, such as interest, dividends, and rents [31]; conversely, a surplus of income over time can contribute to one’s wealth. The distribution of wealth may be as important as the distribution of income in explaining health inequalities [32], but wealth has been less frequently studied, and is less commonly available in administrative or research datasets. Similarly, personal debt appears to be associated with health outcomes [33], but rigorous causal evidence is lacking and data rarely available.

### Public goods

The availability and cost of public goods such as healthcare or social care likely also affects the extent to which income influences health outcomes. A loss of income could more severely limit access to these services in a system that requires payments or insurance cover, compared to one where they are free. Indeed, socioeconomic inequalities in self-reported health and mortality appear to be weaker in countries or regions with well-developed welfare regimes or high expenditure on public goods [34, 35].

### Co-interventions

**Fig. 3:**
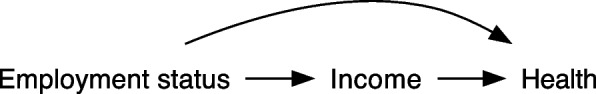
Illustration of how the effect of income on health can be confounded due to causes of income change that also affect health directly

Income changes are frequently the result of events that also affect health directly, as well as via their effects on income: individuals may experience job loss, promotion, childbirth, death of a relative, and so on, while societies may undergo policy changes or natural disasters. For virtually any study, it is crucial to consider how much of any effect is attributable to the income change itself, and how much to the event that caused it. For example, when individuals lose income because of a job loss, a substantial proportion of the effect on mental health appears to be attributable to the job loss itself rather than the income loss [36] (Fig. 3).

Interventions in experimental trials or quasi-experimental evaluations may occasionally consist of a cash transfer and little else, but are frequently delivered together with co-interventions (such as training or non-monetary support) or with conditionality requirements (such as job search requirements or compliance with preventive health measures), which may have a substantial independent effect on many outcomes. Natural experiment studies sometimes rely on specific events that caused income changes (such as a recession or natural disaster), and this makes it very difficult to exclude the possibility that any observed effects are due to the event itself rather than the accompanying changes in income [37].

### Reverse causality

A further challenge is reverse causality: health is an important determinant of income, particularly by affecting one’s ability to work, and hence an income change can be both caused by and the cause of changes in health outcomes [38]. This is a major issue in virtually all observational study designs where the variation in income does not have a random, or at least exogenous, source: although it is most widely acknowledged in the context of cross-sectional studies, it can also be problematic in longitudinal study designs. If both income and health change between two time points, we cannot definitively know which caused which; even if we observe the income change first, we often cannot exclude the possibility that an earlier, unmeasured health factor or event was in fact the cause of both. Natural experiments and instrument-based study designs are particularly important for overcoming this problem, but are relatively underused in public health research [39].

### Applied examples

To illustrate the practical application of our taxonomy, we will consider four studies investigating the effect of income on mental health using contrasting approaches (Table 2): a randomised trial of a conditional cash transfer scheme in New York City (study A) [40]; a natural experiment study based on the introduction of an unconditional cash transfer from casino revenue in North Carolina (study B) [41]; a fixed-effects panel study using Finnish administrative data on earned and unearned income (study C) [42]; and a study exploiting random lottery wins in a Swedish sample as a natural experiment (study D) [43]. We selected these studies to represent some of the most important causal identification strategies in this literature: randomised trial, longitudinal fixed-effects, and a policy-based and a non-policy-based natural experiment. In each of these categories, we selected a study identified in recent systematic reviews as well conducted [5, 6]. We prioritised diversity of study design over homogeneity in outcome measure, but all studies use outcomes that can be seen as proxies for general mental health. We will see that each study has made substantively different choices about the measurement and causal contrast, some at the researchers’ discretion, and some enforced by the choice of study design. These choices crucially affect the meaning of the resulting estimates, and the extent to which the studies can be meaningfully compared or generalised.

**Table 2:** Characteristics of four studies investigating the effects of income on mental health, illustrating the diversity of income measures, causal contrasts, and potential for confounding and effect modification encountered in the literature

	Courtin 2018^a^	Akee 2018^b^	Junna 2019^c^	Lindqvist 2020^d^
Study design	Randomised trial	Natural experiment	Fixed-effects (within-person changes)	Natural experiment
Outcome	Kessler Psychological Distress scale (continuous measure)	Behavioural and emotional disorder symptoms (interviewer-administered questionnaire; continuous measure)	Psychotropic drug purchases (dichotomous measure)	Psychological distress scale (GHQ-12, continuous measure)
Results	Negligible beneficial effect	Beneficial effect	No effect	Small beneficial effect
**Income measure**				
Source	Unearned (specific benefit scheme)	Unearned (specific cash transfer)	Taxable income (earned and some unearned)	Unearned (lottery win)
Gross/net	Gross	Gross	Gross	Net of taxes
Unit of analysis	Household	Household	Individual	Individual
Absolute/relative	Absolute	Absolute	Absolute	Absolute
**Causal contrast**				
Amount	Mean $8,674 in total	~ $6,000 semiannually	Not reported	~ $7 K to $800 K
Transformations/functional form assumptions	Dichotomised exposure (compared increase vs. no increase)	Dichotomised exposure (receiving vs. not receiving transfer)	Log-transformed income	Assumed linear relationship
Direction of change	Increases only	Increases only	Increases and decreases (effects assumed to be symmetric)	Increases only
Duration of change	36 months (time-limited programme)	Cash transfers expected to continue permanently	Year-on-year change in annual income	Single, one-off cash transfer (lottery win)
Timing of exposure and follow-up	Outcome measured 18 months after start of exposure	Exposure at age 13 or 15 years; outcome measured at age 16 years	Outcome measured in the year following income change	Outcome measured 5–22 years after exposure

^a^Courtin E, Muennig P, Verma N, et al. Conditional cash transfers and health of low-income families in the US: evaluating the family rewards experiment. Health Aff. 2018;37(3):438–46

^b^Akee R, Copeland W, Costello EJ, Simeonova E. How does household income affect child personality traits and behaviors? Am Econ Rev. 2018;108(3):775–827

^c^Junna L, Moustgaard H, Tarkiainen L, Martikainen P. The association between income and psychotropic drug purchases: individual fixed effects analysis of annual longitudinal data in 2003–2013. Epidemiology. 2019;30(2):221–29

^d^Lindqvist E, Östling R, Cesarini D. Long-run effects of lottery wealth on psychological well-being. Rev Econ Stud. 2020;87(6):2703–26

Considering the definitions of the income measures, perhaps the most salient difference is that each study concerns a substantially different income source. Although both studies A and B concerned transfers of unearned income, the former was conditional and targeted low-income families, while the latter was unconditionally given to all households in a community. Study C instead looked at total taxable income, a large proportion of which would have been earned, while study D used lottery wins, a very specific and unusual type of unearned income. The use of individual income in study C is a potentially consequential decision, and might underestimate the financial resources of individuals who rely on the income of other household members. Accordingly, the authors conducted additional analyses using household income instead of individual income.

The causal contrast is most clearly defined in the studies with a well-defined intervention (A, B, and D), which looked at the effect of receiving versus not receiving a specified amount of additional income. In contrast, study C used all year-on-year changes in taxable income, and therefore both the amount and reason for change are unknown: or, to put it differently, the exposure is a mixture of many different amounts of and reasons for income change. Only the analyses in studies C and D require explicit functional form assumptions, since studies A and B dichotomised the exposure as either receiving or not receiving the intervention. In study D, the functional form assumptions become particularly important, since the lottery wins studied included very large amounts, in many cases orders of magnitude greater than the median income, and considerably greater than the size of the cash transfers in the other studies. It is perhaps questionable whether these quantitative effect estimates can be meaningfully compared when the exposures are so different. However, insofar as we might try to compare them, the comparison hinges on whether the effect of a massive income gain has a straightforward (say, log-linear) relationship with the effect of a more modest one.

The duration of the income changes varies widely: the cash transfer scheme in study A was a time-limited pilot, and the lottery wins in study D largely one-off windfalls; only study B represents a change that participants may plausibly have seen as stable in the long term. Study C, again, illustrates the challenge in clearly stating the causal contrast when the exposure is within-person changes broadly defined: these may be a mixture of temporary and permanent, expected and unexpected changes, but the analysis cannot distinguish these.

Follow-up time also varies widely: the exceptionally long follow-up of study D is a consequence of its unusual identification strategy (exploiting the intrinsic randomisation of a lottery) and its use of administrative data. Studies A and C are more representative of a large proportion of the existing literature, where follow-up length is limited by practical or methodological considerations: in the former, attrition and the costs of running a formal trial, and in the latter, the limitations of the identification strategy (since confounding and other biases would gradually drown out the true effect if the follow-up time were increased).

With these differences made explicit, it becomes clear that we should not expect the results of such disparate studies to converge on any single answer – even when we only consider the issues of income definition and causal contrast, and no other contextual factors that we would expect to cause additional heterogeneity. This exercise thus underscores the importance for studies to report the exposure clearly so that relevant distinctions are clear to readers [44]. We can also note that among these examples, study B was the only instance of a stable, permanent income increase, and the only study where exposure occurred during childhood. As previously discussed, there are sound theoretical reasons to expect effects on health in those circumstances to be larger. In contrast, much of the existing literature concerns relatively short-term effects of temporary income changes in adult populations [6].

## Concluding remarks

Income as an epidemiological exposure is not a single, well-defined construct. The definitions of “income” and “income change” that any given study uses are not just pragmatic methodological choices, but fundamentally affect what causal pathways we can expect to be involved. Thus, we should expect to find different “causal effects of income” depending on the definitions adopted, even within unbiased studies of the same population. This source of heterogeneity has often been ignored, but has been increasingly highlighted in recent research [5, 7].

Causal inference literature generally holds that causal effects can only be estimated for exposures that are *consistent*, i.e., that do not occur in multiple variations with different causal effects [45]. It can be argued that income as an exposure violates this consistency criterion in many practical applications [46, 47]. However, income is not unique in this respect. A strong argument can be made that no epidemiological exposure satisfies the consistency assumption in the strictest sense, and rather that variations in some aspect or other can always be identified [47]. It is up to the researcher to determine which kinds of variation are problematic. Rather than simply trying to minimise consistency violations, it is perhaps more important to be clear and explicit about what different kinds of exposures and causal contrasts an estimate incorporates, how they are likely to differ, and how these differences are likely to affect the result.

The taxonomy presented here can be used to assess how far effect estimates from a given study are applicable in a given context, to clarify systematically why they might not apply, and hence to identify evidence gaps that need to be addressed. The examples discussed here illustrate that some of the more common types of evidence may be inadequate to understand the effect of persistent long-term income changes, slow-acting pathways, and long-term effects of childhood exposures.

Recent systematic reviews have made progress in explaining heterogeneity in existing evidence using subgroup analysis and meta-regression [5, 6]. We hope this framework will inform and inspire further efforts at evidence synthesis and triangulation, where methodological variety can be harnessed as a source of information rather than seen only as a source of uncertainty [48, 49]. Above all, we encourage researchers to aid in these efforts by being as precise as possible when defining income measures and causal contrasts in future empirical studies.

## Data Availability

No datasets were generated or analysed during the current study.
